# Experimental Validation of Shifted Position-Diffuse Reflectance Imaging (SP-DRI) on Optical Phantoms

**DOI:** 10.3390/s22249880

**Published:** 2022-12-15

**Authors:** Moritz Späth, Alexander Romboy, Ijeoma Nzenwata, Maximilian Rohde, Dongqin Ni, Lisa Ackermann, Florian Stelzle, Martin Hohmann, Florian Klämpfl

**Affiliations:** 1 Institute of Photonic Technologies, Friedrich-Alexander-Universität Erlangen-Nürnberg, 91052 Erlangen, Germany; 2 Erlangen Graduate School in Advanced Optical Technologies, 91052 Erlangen, Germany; 3 Department of Oral and Maxillofacial Surgery, University Hospital Erlangen, 91054 Erlangen, Germany

**Keywords:** SP-DRI, biomedical imaging, all-optical, medical diagnostics, optical phantoms, experimental validation

## Abstract

Numerous diseases such as hemorrhage, sepsis or cardiogenic shock induce a heterogeneous perfusion of the capillaries. To detect such alterations in the human blood flow pattern, diagnostic devices must provide an appropriately high spatial resolution. Shifted position-diffuse reflectance imaging (SP-DRI) has the potential to do so; it is an all-optical diagnostic technique. So far, SP-DRI has mainly been developed using Monte Carlo simulations. The present study is therefore validating this algorithm experimentally on realistic optical phantoms with thread structures down to 10 μm in diameter; a SP-DRI sensor prototype was developed and realized by means of additive manufacturing. SP-DRI turned out to be functional within this experimental framework. The position of the structures within the optical phantoms become clearly visible using SP-DRI, and the structure thickness is reflected as modulation in the SP-DRI signal amplitude; this performed well for a shift along the *x* axis as well as along the *y* axis. Moreover, SP-DRI successfully masked the pronounced influence of the illumination cone on the data. The algorithm showed significantly superior to a mere raw data inspection. Within the scope of the study, the constructive design of the SP-DRI sensor prototype is discussed and potential for improvement is explored.

## 1. Introduction

A strong functional parameter for capturing the state of microcirculation is the so-called functional capillary density (FCD). Per area, this parameter specifies the length of capillaries supplied with blood [1]. It has been shown that there is a relationship between FCD and a poor outcome of a patient [1,2,3]. In this regard, the following holds true: In the physiological state, capillaries in the vicinity of each other are supplied with blood in a quite homogeneous manner. However, this situation reverses in the diseased patient: A heterogeneous perfusion of the capillaries is induced by hemorrhage, sepsis or cardiogenic shock, to name just a few examples. This heterogeneity stems from the fact that capillary perfusion shuts down completely in some tissue areas, while adjacent areas experience only minimal change (ability of vasoconstriction and vasodilation of the capillaries [4,5]). Capillaries supplied with blood and capillaries that are not supplied with blood are thus situated close to each other [2]. These characteristic morphological changes are aimed to be the basis for an identification of the diseases listed above.

To be able to detect the type of alteration in the blood flow pattern described above, diagnostic devices must provide an appropriately high spatial resolution. Microvideoscopic approaches theoretically allow their resolution to be tuned, but they have the disadvantage that they can only be used to a limited extent with critically ill patients (nailfold videocapillaroscopy) or not at all with ventilated patients (orthogonal polarization spectral imaging, sidestream dark field imaging, incident dark field illumination) [2,6,7,8,9]. Confocal laser scanning microscopy has a limited penetration depth and is expensive [10]. Doppler-derived techniques—an important group of clinical diagnostic systems—typically suffer from significant movement artefacts and are therefore not optimal for the flexible clinical use on a patient [11,12,13,14]. The same is true for photoacoustic tomography [15]. The signal of pulse oximetry reflects the situation in larger vessels and is thus not appropriate for the observation of small capillaries [16]. Finally, there is hyperspectral imaging. This technique has a limited resolution which is quantified as approximately 520 μm/pixel for current systems [17]. Note: (Minimally-)Invasive methods will not be discussed at this point as noninvasive approaches are considered as state-of-the-art.

Shifted position-diffuse reflectance imaging (SP-DRI), a noninvasive photonic detection technology invented by our group, has the potential to reach an appropriately high spatial resolution while at the same time overcoming the drawbacks of the systems described above; its imaging depth is with around 0.3 mm sufficiently high [18]. The SP-DRI algorithm leverages light diffusely reflected from the skin to obtain information on the microvasculature located thereunder. It is already subject to a number of scientific investigations conducted and published by us: Monte Carlo (MC) simulations have shown that the technique permits the detection of structures on the order of magnitude of human capillaries within skinlike turbid media [19]. Beyond that, SP-DRI is sufficiently sensitive so that changes in the capillary diameter in the micrometer range can be sensed [20]. This also applies to the case of cross-individual operation (i.e., under nonoptimal conditions) [21]. Experimental results obtained using a liquid optical phantom are available as well [19]. The latter was a proof-of-concept setup in the laboratory.

In this study, a dedicated SP-DRI sensor is introduced that was invented by our group. The different units and the housing of this sensor are explained and its calibration is described. The usability of the SP-DRI sensor is afterwards evaluated by measurements on polydimethylsiloxan (PDMS) phantoms. These phantoms contain structures in the micrometer range; their fabrication is described in the course of the present study.

## 2. Materials and Methods

### 2.1. SP-DRI Sensor

**Figure 1:**
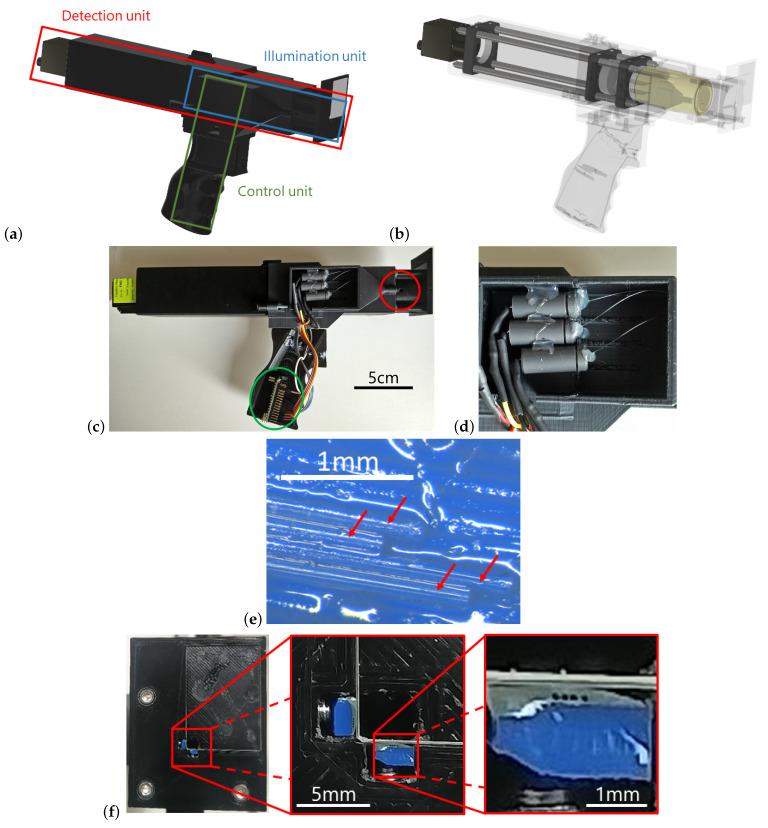
(**a**) CAD illustration of the overall system consisting of an illumination unit and a detection unit and the control of the LEDs via the Arduino Nano. The pistol grip allows for an ergonomic handling of the sensor during measurements. (**b**) By rendering the housing transparent, the optical components of the detection unit become visible. (**c**) Photograph of the side view of the sensor. The cover in the area of the LED light coupling is opened, as is the cover of the Arduino Nano (green circle). The optical fibers are guided into the head of the sensor (red circle). The coupling of the remaining three fibers is located on the opposite side of the sensor. (**d**) Detailed view of the LED light coupling. The housemade sockets can be seen, each of which contains an LED and a ball lens and from which the optical fibers lead out. (**e**) FDM auxiliary construction to hold the distal fiber ends (view parallel to fibres). The fibers are glued into the grooves with two-component epoxy adhesive. The block is then afterward polished from the front as described in the main text. The image shows four optical fibers (marked by red arrows), but only three of them were in use. (**f**) Front of the sensor with the cover glass. The first zoomed-in section of the image displays the aperture through which the detection optics are focused on the sample under investigation, as well as adjacent to that the two illumination fiber blocks. The second zoomed-in section shows the end of a fiber block, the four glued-in and polished fibers are clearly visible.

According to the SP-DRI principle, the sensor consists of an illumination unit and a detection unit. Both are integrated into an additively manufactured housing. A general view of the system can be found in Figure 1a.

#### 2.1.1. Detection Unit

The detection unit takes up the most space in the overall system. It is a traditional microscope configuration consisting of an infinity-corrected objective (5x, Mitutoyo, Kanagawa, Japan) and a tube lens (f = 150 mm) for imaging on a monochrome camera chip (Blackfly S BFS-U3-122S6M-C, FLIR Systems, Wilsonville, OR, USA) recording at a color depth of 16 bit. With the components mentioned, the magnification of the system is 3.75. The detection unit is installed in the interior of the sensor housing; the components are illustrated in Figure 1b.

#### 2.1.2. Illumination Unit

A key aspect of SP-DRI is the required illumination shift. Constructionally, this can be implemented using optical fibres. In the case of the present study, fibers with a core diameter of 50 μm were used (FG050UGA, Thorlabs, Bergkirchen, Germany). In total, eight fibers were needed; at their distal ends, they were shortened to an appropriate length, stripped at their ends and then cleaved. They were then assembled into two blocks of four fibers each in order to subsequently bring them as close as possible to the detection image area of the sensor (see Section 2.1.3 and Figure 1f).

In each block, the glass fibers were arranged in a line in a fixed spacing of 160 μm next to each other. For this purpose, the characteristic layer lines of Fused Deposition Modeling (FDM) prints were used and an auxiliary construction was additively manufactured. The layer thickness was set to 160 μm accordingly. Four fibres were glued into four adjacent grooves of such a block using two-component epoxy adhesive; a photograph of this can be found in Figure 1e. Both blocks were then polished from the front side to achieve an optimal coupling into the medium under investigation later on.

Light from light-emitting diodes (LEDs) with a wavelength of 430 nm was coupled into the proximal tips of the glass fibres. Each fiber is connected to an individual LED so that it can be controlled by which fiber the object under investigation is irradiated at a certain point in time. Per LED, the coupling was effected using a ball lens made from BK7 with a diameter of 6 mm, and the alignment was such that the LED body was in direct contact with the ball lens and the latter in direct contact with the fiber. The three elements were placed on axis. A suitable socket to enable this layout was manufactured in-house. This is depicted in Figure 1d. Only three of the four available fibers per fibre block were connected to LEDs; the remaining fiber was left as backup.

The power supply as well as the control of the individual LEDs is done by means of an Arduino Nano. Its position can be seen in Figure 1c.

#### 2.1.3. Housing

**Figure 2:**
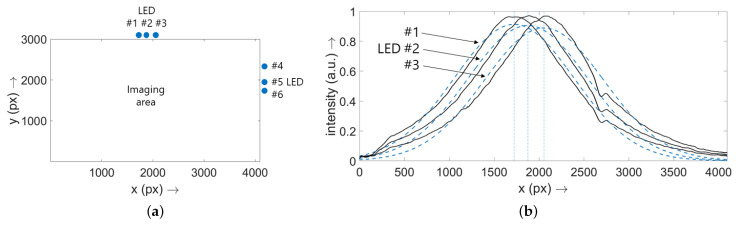
(**a**) Sketch to visualize the location of the imaging area of the SP-DRI sensor and the single LEDs (numerical values are given in Table 1). The view taken here is from the direction of the camera (in contrast to Figure 1f). To achieve a joint origin of both axes of the plot, the y-axis was flipped. This format was also chosen for the results section of this study. Note: Only six LEDs are shown because, as described, one LED per illumination block is kept as backup. (**b**) Exposure situation for the three LEDs running along the *x* axis for the phantom with a thread diameter of 10 μm. The single curves (black) were generated one after the other by placing the sensor on the phantom and turning on the respective LED. A cross-sectional plane at y=3000 px through the recorded diffuse reflectance image was then taken. The Gaussian curves fitted to the raw data are shown as blue dashed lines ($R^{2}>0.96$ for all cases), the maxima of which were interpreted as the *x* positions of the LEDs. The procedure for the LEDs aligned along the *y* axis was identical. As expected, the same values resulted for the second phantom. Note: (**b**) is scaled to 1.

The housing was designed using CAD software and then additively manufactured with an FDM printer. This enabled optimal adaptation to and integration of the functional components introduced above. Special characteristics of the housing are as follows: (1) A plane-parallel glass plate was integrated into the front of the sensor so that the sensor could be placed on the object under investigation in a defined manner. That side of the glass plate facing toward the sample coincides with the working plane of the microscope objective described in Section 2.1.1 (the alteration of the optical path length due to the refractive index of the glass was taken into account accordingly)—in other words: The detection system focuses on the sample surface. On two sides of this glass plate and in the direct vicinity of the detection aperture, the two illumination fiber blocks were mounted (see Figure 1f and Figure 2a). (2) For fine adjustment and focusing of the detection optics, the mounting of the glass plate is attached adjustably to the sensor main housing via O-rings. (3) To allow for comfortable handling of the sensor, an ergonomically designed grip is mounted on the sensor housing at its center of gravity (grip based on [22]).

### 2.2. System Calibration

The coupling of the LED light into the optical fibers varies in quality in each of the single illumination strands. This becomes apparent when measuring the light intensity available at the sensor head. For quantification, the measuring device (PM100D with S130C, Thorlabs) was placed directly in front of the fiber blocks at the sensor head and the strands were switched on one after the other. Table 1 lists the measured values.

To compensate for these differences in the illumination intensity, the exposure time of the camera was adjusted accordingly. The aim was to achieve in the brightest region of each image (i.e., around the respective illumination fiber) pixels with pixel intensity values of approximately 95% of the maximum saturation. This calibration was carried out once for each of both phantoms (details on the phantoms to follow in the subsequent section). The resulting exposure times are given in Table 1.

In order to define the positions of the LEDs along the edges of the imaging field of the SP-DRI sensor, the sensor was placed on a phantom and the LEDs were switched on serially. Cross-sectional planes were then generated at y=3000 px (LEDs along the *x* axis) or x=4096 px (LEDs along the *y* axis), respectively. Gaussian curves were then fitted to these curves to obtain the positions of the single LEDs. The procedure is demonstrated graphically in Figure 2b and the resulting values are listed in Table 1. As expected, the same values resulted also for the other phantom. Note: Although the light cones exhibit an exponential decay, the Gaussian fit is sufficient to determine the LED positions; $R^{2}>0.96$ is true for all cases.

The lateral resolution of the sensor is at least 2.19 μm. This corresponds to the smallest element of a standard USAF target.

### 2.3. Preparation of Optical Phantoms

The optical phantoms used were manufactured in-house. The matrix consists of two-component PDMS (RTV615, Momentive, Waterford, NY, USA). Black ink (Fount India, Pelikan, Switzerland) was used as absorber, while scattering was realized by adding TiO${}_{2}$ with an average particle size of 200 nm (NO-0051-HP, Iolitec, Heilbronn, Germany). The mass fractions were chosen to match the optical properties of the epidermis at 424 nm. For phantom preparation, both additives were initially mixed with the A component of the PDMS and then refined in an ultrasound bath. Subsequently, the PDMS B component was added and the mixture was degassed using a vacuum pump. After insertion of the microstructure (see paragraph below), the material was cured overnight in an oven at 60 degrees Celsius.

Surgical thread with a diameter of 10 μm (Dafilon 11/0, B. Braun, Germany) and 20 μm (Dafilon 10/0, B. Braun, Germany) was used to mimic human microvasculature. This suture material is made from polyamide 6.6, black colored and hence optically absorbent. To mimic the capillary loop pattern, the thread was tightly wrapped around a cannula, fixed and stored overnight in an oven at 70 degrees Celsius. This allowed the thread to take on the loop pattern. Prior to introducing the thread into the readily prepared phantom matrix, it was pulled off the needle and then placed on top of the matrix. The optical phantom was then immediately brought to the oven. Due to gravity, the thread begins to submerge into the matrix, promptly being fixed in a position close below the phantom surface due to the onset of the curing process. For the results presented in this article, with each of the two wires, one final phantom was made (i.e., two final optical phantoms in total).

The depth of the thread structure within the optical phantom was estimated using a reflected light microscope. For this, it was first focused on the surface of the phantom and then, in a second step, on the thread inside the phantom. The resulting traverse path *t* was registered. Taking into account the refractive index of the phantom material $n_{2}$ as well as the numerical aperture of the microscope objective NA, the depth of the structure $d_{structure}$ can be calculated as

(1) $$d_{structure}=\frac{t}{\left(n_{2}-\sqrt{n_{2}^{2}-{NA}^{2}}\right)/\left(n_{1}-\sqrt{n_{1}^{2}-{NA}^{2}}\right)}$$

with $n_{1}=1$ being the refractive index of air [23,24,25].

The depth of the thread was measured several times for both phantoms, and in general, it agreed very well with human anatomical conditions. Nevertheless, it cannot be completely avoided that the thread structure lies above or below this level in some areas. For the measurements in the present publication, it was therefore ensured that—in accordance with the authors’ preliminary research [19,20,21]—only those areas of the phantoms were investigated where the structures are within a depth of 150 μm to 300 μm. The results presented thereafter relate to this accordingly.

### 2.4. Measurement Procedure

To obtain comparable results for the validation of the SP-DRI sensor, it was placed in an auxiliary mounting construction in the laboratory. In addition to a stable and invariable positioning, this auxiliary construction allows the optical phantom to be illuminated from below with white light. In this way, it is possible to ascertain prior to the actual SP-DRI measurements that there is in fact a thread structure—more precisely: a thread structure within the appropriate depth range as described in Section 2.3—within the optical phantom in the sensor’s field of view. Furthermore, this transmitted light image can be compared with the SP-DRI reconstruction for validation purposes. For the actual SP-DRI measurements, this light source was not turned on.

For data collection, an LED was first switched on, then an image was taken with the exposure time defined in Table 1 and stored to memory, and finally, the LED was switched off again. This procedure was carried out serially for all available LEDs and both optical phantoms.

### 2.5. Data Processing Using the SP-DRI Algorithm

The concept of the SP-DRI algorithm has already been described multiple times in the literature [19,20,21]. Citing this, the procedure can briefly be explained as follows.

“In a first step, two diffuse reflectance data sets have to be created. One data set (matrix 1) differs from the second data set (matrix 2) in that the light source is slightly shifted in *x* or *y* direction at otherwise identical simulation parameters. Second, the two matrices are shifted against each other so that the relative positions of the light source in both matrices are equal with respect to their *x* and *y* coordinates. This leads to a relative shift of the capillary structures to each other. Finally, one intensity matrix is divided pixelwise by the other one.” [20]

According to the above description, the SP-DRI algorithm was applied to the data generated as per Section 2.4. Exemplarily, for each recording series, the shift from LED #1 to LED #2 was analyzed as well as the shift from LED #4 to LED #5. A 2-D Gaussian filter (standard deviation of smoothing kernel: 5px) was applied to the resulting division matrices. Cross-sectional planes through these data maps are additionally postprocessed with a Savitzky–Golay filter (polynomial order: 5; frame length: 151px).

## 3. Results

**Figure 3:**
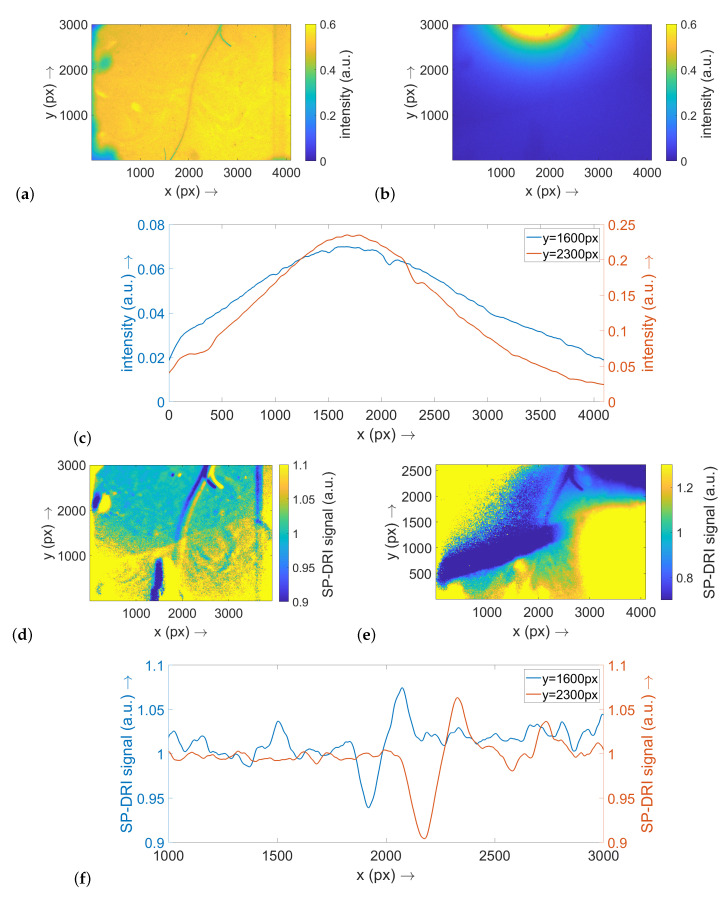
This figure shows the results for the optical phantom for the configuration with a thread of 10 μm diameter. (**a**) Transmitted light image using a broadband light source; this image serves as reference for assessing the data reconstruction presented in the following subimages. (**b**) Exemplary diffuse reflectance raw data set when illuminating the optical phantom with LED #1. (**c**) Cross-sections along the *x* axis through the raw data map shown in (**b**) at the two *y* positions indicated. (**d**) SP-DRI reconstruction based on the diffuse reflectance matrices for the illumination with LED #1 and LED #2 (the matrix shift is thus along the *x* axis). (**e**) SP-DRI reconstruction based on the diffuse reflectance matrices for the illumination with LED #4 and LED #5 (the matrix shift is thus along the *y* axis). (**f**) Cross-sections along the *x* axis through the SP-DRI map shown in (**d**) at the two *y* positions indicated. Note: (**a**–**c**) are scaled to 1.

In the following, the results for the experimental investigations on the two optical phantoms are presented. The data for the phantom with a thread diameter of 10 μm will be described first; they are illustrated in Figure 3. The reference image taken in transmitted light initially shows that there is indeed a thread structure in the imaging area covered by the SP-DRI sensor (Figure 3a). The blurriness in the visualization of the thread in the centre of this image indicates that the structure in this region is positioned deeper within the phantom than in the peripheral regions. Therefore, statements about variable structure depths can be made in the following.

Considering the exemplary diffuse reflectance raw data, the relatively strong light cone around the illumination LED is particularly notable (Figure 3b). The thread structure is only partially perceivable. The two displayed cross-sectional planes along the *x* axis—one at a *y* position in the center of the image and one at its periphery—illustrate this and prove that the thread texture is only weakly reflected in the diffuse reflectance signal (Figure 3c).

The SP-DRI algorithm was applied once along the *x* axis (illumination shift from LED #1 to LED #2; Figure 3d) and once along the *y* axis (illumination shift from LED #4 to LED #5; Figure 3e). As the thread in this example is fairly oriented along the *y* axis, the SP-DRI reconstruction with the shift perpendicular to this axis is particularly useful for detecting the thread structure. Once again, corresponding cross-section planes were prepared (at the same positions as previously described for the raw data; Figure 3f). From these, it can be deduced that the position of the thread structure can be clearly identified using SP-DRI: A significant deflection is evident in the SP-DRI signal, and its position is consistent with the position that is to be expected from the transmitted light reference image.

**Figure 4:**
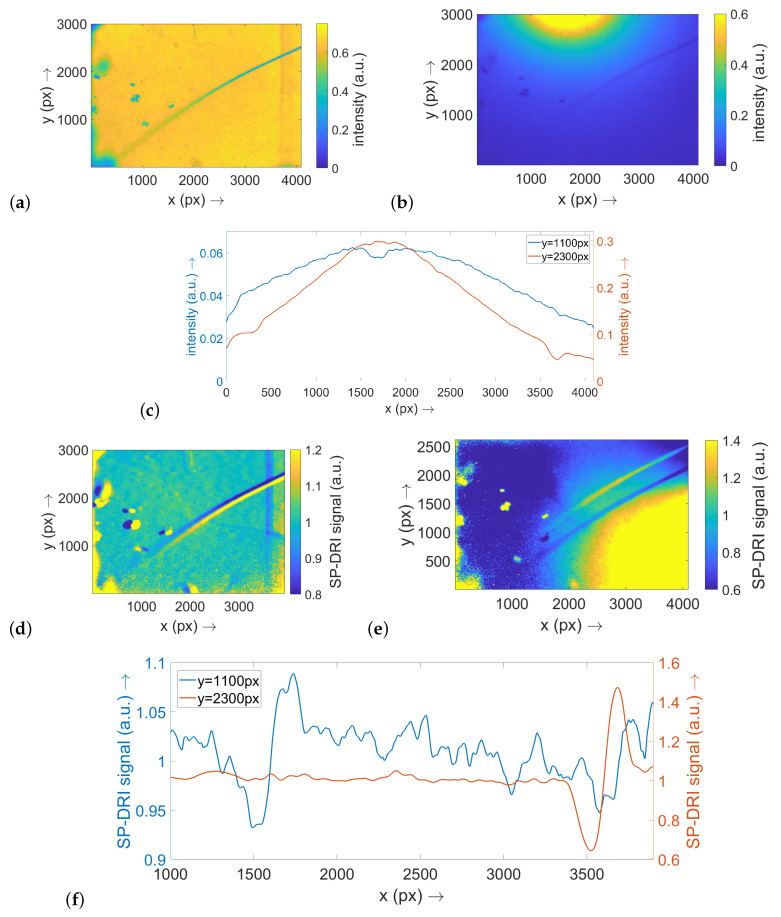
This figure shows the results for the optical phantoms for the configuration with a thread of 20 μm diameter. As the single subillustrations are systematically corresponding to Figure 3, their description can be looked up there accordingly. Note: (**a**–**c**) are scaled to 1.

For the optical phantom with a thread diameter of 20 μm, an analogous statement can be made. In this case, the thread appears in focus on the right-hand side of the image, while its representation becomes considerably blurred in the center and even more on the left-hand side of the image. The more pronounced blurring compared to the previous situation (compare Figure 3a and Figure 4a) indicates that the thread is already very deep within the phantom in the left part of the image. Again, the structure is only weakly visible in the raw data map (Figure 4b) and in the corresponding cross-sectional planes (Figure 4c), while the SP-DRI signal shows a clear deflection (Figure 4f). Since the thread in this example is not primarily aligned along one image axis but runs approximately diagonal thereto, a matrix shift along the *x* axis (Figure 4d) as well as a shift along the *y* axis (Figure 4e) produces a clear signal.

## 4. Discussion

In the present study, the SP-DRI algorithm was evaluated on realistic optical phantoms. Since the aim of this diagnostic approach is to detect changes in the diameter of human capillaries in the micrometre range, phantoms with a sufficiently thin structure had to be made. Surgical thread with diameters of 10 μm and 20 μm was utilized for this purpose. Its black color is highly absorbent, similar to haemoglobin at the selected illumination wavelength of 430 nm.

It can be stated first of all that the SP-DRI algorithm is functional in both phantom configurations: The position of the thread can be determined successfully both close to the phantom surface and deeper inside the phantom. SP-DRI is clearly superior to simply inspecting the raw data images: Although slight changes in the diffuse reflectance curves may also be suspected in the case of the raw data, these changes are significantly weaker (in terms of percentages) than the SP-DRI signal deflections. In this respect, the experimental data confirm the findings about the general functionality of the algorithm which has been shown previously by MC simulations [19]. This also highlights once again the superiority in the performance of SP-DRI over microvideoscopic approaches and techniques such as photoacoustic tomography, pulse oximetry, or hyperspectral imaging, the disadvantages of which have already been described in detail in the introductory chapter of this study. Other approaches based on diffuse reflection described in the literature also cannot match the performance of SP-DRI, in particular, the lateral resolution achieved there is below that of SP-DRI; in some cases, imaging is possible only in transmission mode [26,27,28,29].

Simulatively, it could also be shown that a larger capillary diameter results in a higher amplitude of the SP-DRI signal [20]. To validate this finding experimentally, two different thread diameters were employed in the context of the present study. Looking at the cross-sectional planes through the SP-DRI signal maps for the two regions where the thread is close to the phantom surface, it is evident that the signal generated by the 20 μm diameter threads is significantly stronger than that generated by the 10 μm diameter thread. Therefore, this simulative observation can be regarded as experimentally validated.

A comparison of the SP-DRI signals from the phantom regions where the thread is lying deeper also supports this conclusion: As already described in the results section, it must be assumed that the thread with a diameter of 20 μm (in the examined image area) has sunk deeper into the phantom than the thread with a diameter of 10 μm. However, the corresponding SP-DRI curves show a quantitatively comparable deflection—as deeper structures also result in a lower amplitude [18], the wire thickness must therefore have compensated for the wire depth, which is in line with expectations.

In general, it can be stated that a functional SP-DRI sensor prototype was developed within the framework of the present study. The simulative investigations already mentioned formed the basis for the conceptual design of the sensor. Especially along the long side (*x* axis) of the imaging field, the linear arrangement of the LEDs as well as the adjustment of the image saturation worked well—it can be seen in Figure 3d and Figure 4d that the SP-DRI reconstruction is not superimposed by the intensity cone around the single LEDs. Along the short side (*y* axis), however, there is still space for improvement. Note: Although the raw data curves may exhibit a higher signal-to-noise ratio than the SP-DRI data curves, the latter emphasise the thread structure far more effectively due to the excellent reduction of the background achievable with the SP-DRI algorithm.

The handling and assembly of the single optical fibers proved to be the most difficult part of the setup, and optimizations might still be possible. One simple option, for example, would be to procure and install preassembled fiber blocks. To miniaturize the setup, a fibre optic plate (FOP) could be used, which is placed in direct contact with the camera chip on one side and the object under investigation on the other side. Unfortunately, this approach is accompanied by a reduction in lateral resolution.

It should be emphasized once again at this point that the presented measurements are initial validation results; in particular, the data processing steps were not yet automated. Nevertheless, SP-DRI was clearly functional. It is to be expected that more elaborate data processing techniques (including abandoning the assumption that the single LEDs are arranged perfectly collinear to the imaging field, i.e., rather taking into account both axes at a time when performing the matrix shift) would provide even better reconstruction results. The adjustment of the exposure could also be automated and the entire image area could be taken into account to enable a finer calibration. For the proof-of-concept sensor, however, attention was paid especially to the constructive aspect. Not least, the high lateral resolution achieved proves that the alignment of all components performed excellently.

In addition to further optimising the SP-DRI sensor, a next step is to apply the method ex vivo. The results of the present study are encouraging for a successful application of the presented diagnostic technique in this modality.

## 5. Conclusions

The present study evaluated the SP-DRI algorithm on realistic optical phantoms with embedded structures on the order of magnitude of 10 μm. It was found that the SP-DRI algorithm is functional and clearly superior to simply inspecting the raw data images.

With SP-DRI, the position of the structures within the optical phantoms could be determined successfully. Moreover, it could be shown that a larger diameter of the embedded structure results in a higher amplitude of the SP-DRI signal. This way, the results obtained by simulations in earlier studies could be validated experimentally.

Although the SP-DRI sensor has been successfully validated, there is room for improvement. The present study provides definite indications for this; they can be found in particular in the underlying software and the use of optical fibers.

## Figures and Tables

**Table 1:** Technical and constructional data of the illumination used. The exposure time refers to the camera, not to the duration that the LEDs were switched on. The position of the LEDs is further illustrated in Figure 2a. px = pixel.

LED	Position Regarding Camera Sensor	Intensity (μW)	Exposure (ms) for 10 μm Phantom	Exposure (ms) for 20 μm Phantom
#1	long side (x=1722px)	12.0	115	200
#2	long side (x=1875px)	5.1	270	480
#3	long side (x=2055px)	0.7	1900	3450
#4	short side (y=2336px)	12.4	180	270
#5	short side (y=1947px)	1.6	1300	1900
#6	short side (y=1736px)	5.7	380	540

## Data Availability

The data presented in this study are available on request from the corresponding author.

## Abbreviations

The following abbreviations are used in this manuscript:

FCD	Functional capillary density
FDM	Fused Deposition Modeling
MC	Monte Carlo
PDMS	Polydimethylsiloxan
px	pixel
SP-DRI	Shifted position-diffuse reflectance imaging
